# Efficacy and safety of switching to dolutegravir/lamivudine in virologically suppressed people with HIV-1 aged ≥ 50 years: week 48 pooled results from the TANGO and SALSA studies

**DOI:** 10.1186/s12981-024-00604-9

**Published:** 2024-03-21

**Authors:** Sharon Walmsley, Don E. Smith, Miguel Górgolas, Pedro E. Cahn, Thomas Lutz, Karine Lacombe, Princy N. Kumar, Brian Wynne, Richard Grove, Gilda Bontempo, Riya Moodley, Chinyere Okoli, Michelle Kisare, Bryn Jones, Andrew Clark, Mounir Ait-Khaled

**Affiliations:** 1 University Health Network, 200 Elizabeth Street, Toronto, ON M5G 2C4 Canada; 2 Albion Centre, 150 Albion Street, Surry Hills NSW 2010, Sydney, Australia; 3 Fundación Jiménez Díaz, Universidad Autónoma de Madrid, Av. de los Reyes Católicos, 2, 28040 Madrid, Spain; 4 Fundación Huésped, Dr. Carlos A. Gianantonio 3932, C1204 CABA Buenos Aires, Argentina; 5 Infektiologikum, Stresemannallee 3, 60596 Frankfurt am Main, Frankfurt, Germany; 6 Hôpital Saint-Antoine, 184 Rue du Faubourg Saint-Antoine, 75012 Paris, France; 7 Georgetown University Medical Center, 4000 Reservoir Road, NW, Washington, DC 20057 USA; 8 ViiV Healthcare, 406 Blackwell Street, Suite 300, Durham, NC 27701 USA; 9 GSK, 980 Great West Road, Brentford, Middlesex TW8 9GS UK; 10 ViiV Healthcare, 36 E Industrial Road, Branford, CT 06405 USA; 11 ViiV Healthcare, 980 Great West Road, Brentford, Middlesex TW8 9GS UK

**Keywords:** Aging, Comorbidity, DTG/3TC, HIV-1, Polypharmacy, Single-tablet regimen, Suppressed switch, ≥ 50 years

## Abstract

**Background:**

As the population of people with HIV ages, concerns over managing age-related comorbidities, polypharmacy, immune recovery, and drug-drug interactions while maintaining viral suppression have arisen. We present pooled TANGO and SALSA efficacy and safety results dichotomized by age (< 50 and ≥ 50 years).

**Methods:**

Week 48 data from the open-label phase 3 TANGO and SALSA trials evaluating switch to once-daily dolutegravir/lamivudine (DTG/3TC) fixed-dose combination vs continuing current antiretroviral regimen (CAR) were pooled. Proportions of participants with HIV-1 RNA ≥ 50 and < 50 copies/mL (Snapshot, intention-to-treat exposed) and safety were analyzed by age category. Adjusted mean change from baseline in CD4 + cell count was assessed using mixed-models repeated-measures analysis.

**Results:**

Of 1234 participants, 80% of whom were male, 29% were aged ≥ 50 years. Among those aged ≥ 50 years, 1/177 (< 1%) DTG/3TC participant and 3/187 (2%) CAR participants had HIV-1 RNA ≥ 50 copies/mL at 48 weeks; proportions with HIV-1 RNA < 50 copies/mL were high in both treatment groups (≥ 92%), consistent with overall efficacy and similar to observations in participants aged < 50 years (≥ 93%). Regardless of age category, CD4 + cell count increased or was maintained from baseline with DTG/3TC. Change from baseline in CD4 + /CD8 + ratio was similar across age groups and between treatment groups. One CAR participant aged < 50 years had confirmed virologic withdrawal, but no resistance was detected. In the DTG/3TC group, incidence of adverse events (AEs) was similar across age groups. Proportions of AEs leading to withdrawal were low and comparable between age groups. Although drug-related AEs were generally low, across age groups, drug-related AEs were more frequent in participants who switched to DTG/3TC compared with those who continued CAR. While few serious AEs were observed in both treatment groups, more were reported in participants aged ≥ 50 years vs < 50 years.

**Conclusions:**

Among individuals with HIV-1, switching to DTG/3TC maintained high rates of virologic suppression and demonstrated a favorable safety profile, including in those aged ≥ 50 years despite higher prevalence of concomitant medication use and comorbidities.

*Trial registration number:* TANGO, NCT03446573 (February 27, 2018); SALSA, NCT04021290 (July 16, 2019).

**Supplementary Information:**

The online version contains supplementary material available at 10.1186/s12981-024-00604-9.

## Background

The availability of highly effective antiretroviral therapy (ART) has resulted in life expectancy for people with HIV similar to that of the general population [1, 2]. Further, the number of people aged ≥ 50 years acquiring HIV, who are often diagnosed later in the disease course, has increased [2]. As a consequence, globally, the proportion of individuals with HIV aged ≥ 50 years rose from 8% in 2000 to 16% in 2016 and to an estimated 24% in 2022 [3–5]. In 2022, in Western and Central Europe and North America, nearly half of all individuals with HIV were aged ≥ 50 years [5]. Predictive modeling has estimated that by 2030, 70% of people with HIV in the United States will be aged > 50 years [6], and ~ 25% will be aged > 65 years [7]. Similarly, predictive modeling using cohorts from the Netherlands and France has estimated that the population of people with HIV aged ≥ 50 years will reach 73% and > 66%, respectively, by 2030 [8, 9]. However, although individuals aged ≥ 50 years are among the fastest growing segment of the population with HIV [10], they have historically been underrepresented in or excluded from HIV clinical trials [11, 12].

As people with HIV age, treatment requirements to support healthy living extend beyond achieving and maintaining virologic suppression to also managing age-related comorbidities, polypharmacy, and other healthcare priorities. Therefore, addressing the treatment needs of individuals aged ≥ 50 years while maintaining virologic suppression may present additional considerations compared with treatment of those aged < 50 years [13]. Evidence suggests that people with HIV have increased rates and earlier presentation of comorbidities compared with those without HIV, and prevalence of comorbidities increases with age [6, 14, 15]. Additionally, studies have demonstrated more frequent use of concomitant medications as individuals with HIV age [15, 16], which may result in significant drug-drug interactions with ART. Moreover, ART adherence, and consequently viral suppression, have been negatively associated with polypharmacy [17], particularly in individuals aged ≥ 50 years [18, 19]. Therefore, evaluating the efficacy, safety, and tolerability of ART in people with HIV as they age is critical.

Some of the challenges associated with polypharmacy and increased use of concomitant non-ART medications may be alleviated by using single-tablet or 2-drug ART regimens instead of multi-tablet or 3- or 4- drug regimens. Dolutegravir/Lamivudine (DTG/3TC) is a 2-drug, single-tablet regimen that is recommended in international guidelines and has demonstrated durable efficacy, a high barrier to resistance, and good safety and tolerability as first-line ART in treatment-naive participants in the phase 3 GEMINI-1 and GEMINI-2 trials and as a switch option in virologically suppressed participants in the phase 3 TANGO and SALSA studies [7, 20–22]. Subgroup analyses of TANGO and SALSA showed high efficacy and a good safety profile for DTG/3TC in participants aged ≥ 50 years, but sample sizes were small (TANGO, n = 79; SALSA, n = 98) [23, 24]. Here, we present efficacy and safety of DTG/3TC in a larger sample of participants aged ≥ 50 years by pooling the analyses from the TANGO and SALSA studies.

## Methods

### Study design

This analysis included pooled Week 48 data from the open-label phase 3 TANGO and SALSA trials evaluating switch to once-daily DTG/3TC fixed-dose combination vs continuing current antiretroviral regimen (CAR). Detailed methodology for the TANGO (ClinicalTrials.gov, NCT03446573) and SALSA (ClinicalTrials.gov, NCT04021290) studies has previously been published [20, 21]. In brief, eligibility criteria included no prior virologic failure, no documented nucleoside reverse transcriptase inhibitor (NRTI) or integrase strand transfer inhibitor (INSTI) resistance, no hepatitis B virus infection or need for hepatitis C virus therapy, and documented virologic suppression (HIV-1 RNA < 50 copies/mL for > 6 months). Exclusion criteria included plasma HIV-1 RNA ≥ 50 copies/mL within 6 months of screening or ≥ 2 measurements ≥ 50 copies/mL or any measurement > 200 copies/mL within 6 and 12 months of screening. Pooled data were dichotomized by age categories (< 50 and ≥ 50 years). Data were also summarized in a third category (≥ 65 years), but due to the low numbers of participants, no statistical analyses for this category were conducted.

Both studies were conducted in accordance with the International Conference on Harmonization Good Clinical Practice and followed the principles of the Declaration of Helsinki; all participants provided written informed consent before study initiation.

### Procedures

Detailed procedures have been previously published [20, 21]. Briefly, eligible participants were randomized 1:1 to switch to once-daily DTG/3TC fixed-dose combination or continue their current ART regimen. In TANGO, participants switched from tenofovir alafenamide/emtricitabine (TAF/FTC) plus a protease inhibitor (PI), INSTI, or non-nucleoside reverse transcriptase inhibitor (NNRTI). Participants with initial tenofovir disoproxil fumarate (TDF) treatment who switched to TAF ≥ 3 months before screening, with no changes to other drugs in their regimen, were also eligible. In SALSA, participants on uninterrupted ART for ≥ 3 months switched from regimens composed of 2 NRTIs plus a PI, INSTI, or NNRTI. No regimen modifications were allowed on study, except switching between ritonavir and cobicistat (TANGO, SALSA) or 3TC and FTC (SALSA) in the CAR group. Study visits were planned at baseline (Day 1) and Weeks 4, 8 (TANGO only), 12, 24, 36, and 48. Plasma for HIV-1 RNA quantification was collected and safety outcomes were assessed at each visit.

### Outcomes

The primary endpoint was the proportion of participants with HIV-1 RNA ≥ 50 copies/mL at Week 48 using the US Food and Drug Administration Snapshot algorithm in the intention-to-treat–exposed (ITT-E) population. Key secondary endpoints included proportion of participants with HIV-1 RNA < 50 copies/mL (Snapshot, ITT-E) and proportion of participants meeting confirmed virologic withdrawal (CVW) criteria, defined as HIV-1 RNA ≥ 50 copies/mL followed by a second consecutive on-treatment HIV-1 RNA ≥ 200 copies/mL. Other secondary endpoints included change from baseline in CD4 + cell count and CD4 + /CD8 + ratio. Safety assessments included incidence and severity of adverse events (AEs) as well as change from baseline in weight, lipid parameters, and renal and bone biomarkers.

### Statistical analysis

All randomized participants who received ≥ 1 dose of study treatment were included in the ITT-E population, which was used for efficacy and safety analyses. Proportions of participants with HIV-1 RNA ≥ 50 copies/mL and < 50 copies/mL (Snapshot, ITT-E) at Week 48 were analyzed using a Cochran-Mantel–Haenszel test adjusting for baseline third agent class. Mixed-models repeated-measures analysis was used for adjusted mean change from baseline in CD4 + cell count, CD4 + /CD8 + ratio, weight, lipid parameters, and renal and bone biomarkers in the 2 age groups (< 50 and ≥ 50 years). Adjustment terms included treatment, visit, age, sex, race, baseline value, baseline third agent class, baseline CD4 + cell count, treatment-by-visit interaction, baseline value-by-visit interaction, and study, with visit as the repeated factor; subgroup analyses by age were also adjusted for visit-by-age, treatment-by-age, and treatment-by-visit-by-age interactions. Baseline body mass index (BMI) was an additional adjustment term for CD4 + cell count and CD4 + /CD8 + ratio. Baseline TAF and TDF use were additional adjustment terms for weight. Baseline BMI, diabetes, and hypertension were additional adjustment terms for renal biomarkers. Baseline BMI, smoking history, and vitamin D use were additional adjustment terms for bone biomarkers. If logistic regression models did not converge due to either small sample size or low event rates, a Firth penalized maximum likelihood regression model generating product-limit CIs was used instead [25]. To determine treatment regimen–adjusted likelihood of ≥ 10% weight gain at Week 48, a logistic Firth regression model analysis was used. Adjustment terms were treatment, sex, age, race, baseline third agent class, baseline CD4 + cell count, baseline weight, and study. Incidence and severity of AEs were summarized descriptively.

## Results

### Participants

**Fig. 1:**
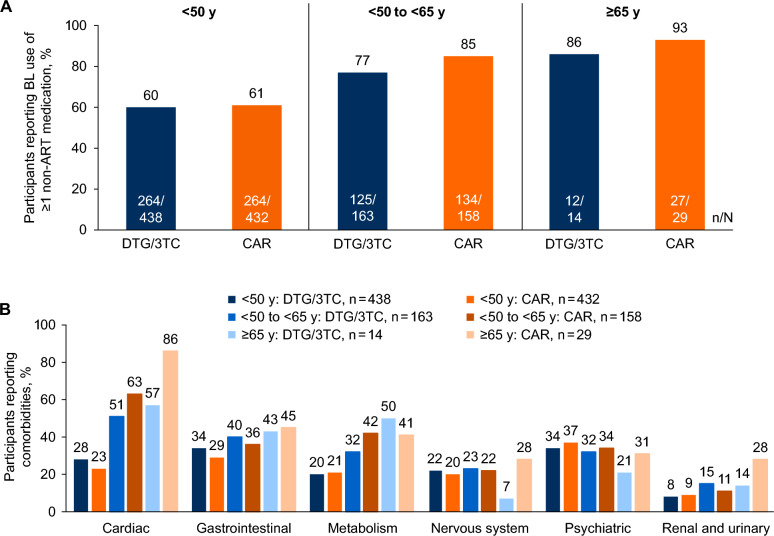
Baseline **A** use of ≥ 1 non-ART medication and **B** comorbidities by age. TANGO and SALSA pooled ITT-E population. *BL* baseline, *CAR* current antiretroviral regimen, *DTG* dolutegravir, *ITT-E* intention-to-treat exposed, *3TC* lamivudine

Of 1234 participants in the pooled TANGO and SALSA ITT-E population (DTG/3TC, n = 615; CAR, n = 619), 71% (n = 870) were aged < 50 years and 29% (n = 364) were aged ≥ 50 years (Table 1). Of note, 3% (n = 43) were aged ≥ 65 years. Overall, baseline characteristics were similar between the DTG/3TC and CAR groups. Baseline characteristics between the < 50 and ≥ 50 years age groups were generally similar across treatment groups. However, participants aged ≥ 50 years had greater concomitant medication use, more comorbidities, and longer prior ART duration compared with participants aged < 50 years. In both treatment groups, the proportions of participants with baseline non-ART medication use and comorbidities increased with age (Fig. 1). Cardiac, gastrointestinal, and metabolism disorders were the most prevalent comorbidities in participants aged ≥ 50 years. Across all participants, 131 (11%) had polypharmacy (use of > 5 medications) at baseline. The most commonly used (> 5% of the overall population) concomitant medications were cholecalciferol (9%), vitamins (not otherwise specified; 8%), and ibuprofen (6%).

**Table 1:** Demographics and Baseline Characteristics Overall and by Age: TANGO and SALSA Pooled ITT-E Population

Parameter	Overall		< 50 y		50 to < 65 y		≥ 65 y	
	DTG/3TC (N = 615)	CAR (N = 619)	DTG/3TC (N = 438)	CAR (N = 432)	DTG/3TC (N = 163)	CAR (N = 158)	DTG/3TC (N = 14)	CAR (N = 29)
Sex, female, n (%)	133 (22)	117 (19)	68 (16)	71 (16)	59 (36)	38 (24)	6 (43)	8 (28)
Age, median (range), y	42 (20–74)	42 (18–83)	37 (20–49)	36 (18–49)	56 (50–64)	55 (50–64)	67 (65–74)	69 (65–83)
Weight, median (range), kg	77 (43–154)	78 (36–160)	78 (43–154)	77 (48–160)	75 (44–128)	80 (36–127)	72 (59–106)	75 (49–116)
BMI, median (range), kg/m^2^	25 (17–51)	26 (14–69)	25 (17–51)	25 (17–69)	25 (18–43)	27 (14–45)	26 (18–32)	27 (19–44)
Baseline use of ≥ 1 non-ART medication, n (%)	401 (65)	425 (69)	264 (60)	264 (61)	125 (77)	134 (85)	12 (86)	27 (93)
Number of baseline non-ART medications, median (range)	1 (0–13)	1 (0–20)	1 (0–12)	1 (0–16)	2 (0–13)	3 (0–20)	2 (0–6)	4 (0–11)
Baseline polypharmacy (> 5 medications), n (%)^a^	55 (9)	76 (12)	—	—	—	—	—	—
Baseline comorbidities, n (%)	457 (74)	474 (77)	311 (71)	307 (71)	134 (82)	138 (87)	12 (86)	29 (100)
Baseline CD4 + cell count, median (range), cells/mm^3^	680 (133–2089)	684 (94–1954)	685 (154–1904)	686 (94–1954)	653 (133–2089)	677 (119–1530)	638 (228–1199)	657 (122–1133)
Baseline CD4 + /CD8 + ratio, mean (SD)	1.1 (0.54)	1.1 (0.50)	1.0 (0.51)	1.0 (0.44)	1.1 (0.61)	1.1 (0.65)	1.2 (0.58)	1.0 (0.46)
Duration of ART before Day 1, median (range), mo	41 (4–240)	45 (7–253)	37 (4–188)	41 (7–206)	61 (7–240)	57 (9–253)	58 (8–201)	74 (20–214)
Baseline NRTI, n/N (%)^b^								
TDF	109/605 (18)	110/606 (18)	70/431 (16)	73/422 (17)	34/160 (21)	31/155 (20)	5/14 (36)	6/29 (21)
TAF	451/605 (75)	462/606 (76)	340/431 (79)	333/422 (79)	104/160 (65)	109/155 (70)	7/14 (50)	20/29 (69)
ABC	45/605 (7)	34/606 (6)	21/431 (5)	16/422 (4)	22/160 (14)	15/155 (10)	2/14 (14)	3/29 (10)
Baseline third agent, n (%)								
INSTI	387 (63)	394 (64)	295 (67)	285 (66)	83 (51)	97 (61)	9 (64)	12 (41)
NNRTI	174 (28)	172 (28)	109 (25)	110 (25)	61 (37)	47 (30)	4 (29)	15 (52)
PI	54 (9)	53 (9)	34 (8)	37 (9)	19 (12)	14 (9)	1 (7)	2 (7)

*ABC* abacavir, *ART* antiretroviral therapy, *BMI* body mass index, *CAR* current antiretroviral regimen, *DTG* dolutegravir, *INSTI* integrase strand transfer inhibitor, *ITT-E* intention-to-treat exposed, *NNRTI* non-nucleoside reverse transcriptase inhibitor, *NRTI* nucleoside reverse transcriptase inhibitor, *PI* protease inhibitor, *TAF* tenofovir alafenamide, *3TC* lamivudine, *TDF* tenofovir disoproxil fumarate

^a^Data available for overall treatment groups only. ^b^Other NRTI backbone regimens were included in SALSA (zidovudine, tenofovir disoproxil succinate, biovir [not otherwise specified])

### Efficacy

**Fig. 2:**
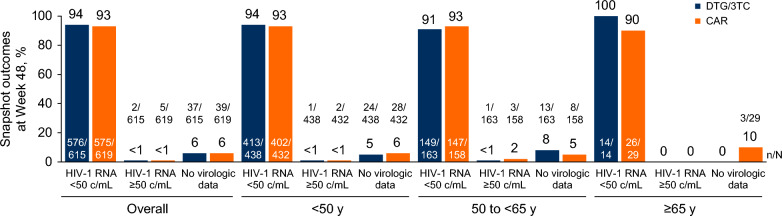
Snapshot virologic outcomes at Week 48 overall and by age. TANGO and SALSA pooled ITT-E population. *CAR* current antiretroviral regimen, *c/mL* copies/mL, *DTG* dolutegravir, *ITT-E* intention-to-treat exposed, *3TC* lamivudine

Proportions of participants with HIV-1 RNA ≥ 50 copies/mL (Snapshot, ITT-E) at Week 48 were similar among all participants regardless of age and across treatment groups (DTG/3TC vs CAR: < 50 years, < 1% [1/438] vs < 1% [2/432]; ≥ 50 years, < 1% [1/177] vs 2% [3/187]), which was consistent with results from the overall study population (DTG/3TC, < 1% [2/615]; CAR, < 1% [5/619]; Fig. 2). Notably, no participants aged ≥ 65 years had HIV-1 RNA ≥ 50 copies/mL in either treatment group. Similarly, proportions of participants with HIV-1 RNA < 50 copies/mL were high across all age and treatment groups (DTG/3TC vs CAR: < 50 years, 94% [413/438] vs 93% [402/432]; 50 to < 65 years, 91% [149/163] vs 93% [147/158]; ≥ 65 years, 100% [14/14] vs 90% [26/29]) and consistent with overall study results (DTG/3TC, 94% [576/615]; CAR, 93% [575/619]). Regardless of age category, no participants in the DTG/3TC group met CVW criteria, while 1 participant aged < 50 years in the CAR group met CVW criteria with no resistance detected.

Across both age and treatment groups, adjusted mean change (SE) in CD4 + cell count from baseline to Week 48 was similar (DTG/3TC vs CAR: < 50 years, 29.0 [8.5] vs 7.6 [8.2] cells/mm^3^; ≥ 50 years, 6.3 [13.6] vs − 24.7 [12.5] cells/mm^3^), which reflected results from the overall study population (DTG/3TC, 22.4 [7.2] vs CAR, − 2.0 [6.9] cells/mm^3^; Additional file 1). Similar results were also observed for adjusted mean change (SE) from baseline to Week 48 in CD4 + /CD8 + ratio (DTG/3TC vs CAR: < 50 years, 0.039 [0.010] vs 0.048 [0.010]; ≥ 50 years, 0.032 [0.016] vs 0.062 [0.016]; overall, 0.037 [0.008] vs 0.052 [0.009]).

### Safety

#### Adverse events

Incidence of AEs was comparable across age and treatment groups (Table 2). Adverse events leading to withdrawal and drug-related AEs were low and comparable between age groups. While serious AEs (SAEs) were generally low, more SAEs were observed in participants aged ≥ 50 years in both treatment groups (DTG/3TC vs CAR: < 50 years, 4% [18/438] vs 4% [16/432]; 50 to < 65 years, 5% [8/163] vs 8% [12/157]; ≥ 65 years, 14% [2/14] vs 14% [4/29]). In all age categories, AE incidence was comparable between the DTG/3TC and CAR groups, although drug-related AEs were more frequent in participants who switched to DTG/3TC compared with those who continued CAR (DTG/3TC vs CAR: < 50 years, 15% [65/438] vs 4% [18/432]; 50 to < 65 years, 15% [25/163] vs 2% [3/157]; ≥ 65 years, 21% [3/14] vs 0% [0/29]), which was consistent with the overall population (DTG/3TC, 15% [93/615]; CAR, 3% [21/618]).

**Table 2:** Adverse Events Through Week 48 Overall and by Age: TANGO and SALSA Pooled Safety Population^a^

Parameter, n (%)	Overall		< 50 y		50 to < 65 y		≥ 65 y	
	DTG/3TC (N = 615)	CAR (N = 618)	DTG/3TC (N = 438)	CAR (N = 432)	DTG/3TC (N = 163)	CAR (N = 157)	DTG/3TC (N = 14)	CAR (N = 29)
Any AE	475 (77)	464 (75)	333 (76)	330 (76)	133 (82)	113 (72)	9 (64)	21 (72)
AEs leading to withdrawal	18 (3)	5 (< 1)	11 (3)	2 (< 1)	7 (4)	2 (1)	0	1 (3)
Grade 2–5 AEs	281 (46)	303 (49)	189 (43)	206 (48)	85 (52)	82 (52)	7 (50)	15 (52)
Drug-related AEs	93 (15)^b^	21 (3)^c^	65 (15)	18 (4)	25 (15)	3 (2)	3 (21)^d^	0
Serious AEs^e^	28 (5)^f^	32 (5)	18 (4)^f^	16 (4)	8 (5)	12 (8)	2 (14)	4 (14)

*AE* adverse event, *CAR* current antiretroviral regimen, *DTG* dolutegravir, *3TC* lamivudine, *TDF* tenofovir disoproxil fumarate

^a^In TANGO, 1 participant was found to be taking a TDF-based regimen and was excluded from the safety population. ^b^The most common (≥ 0.5%) grade 2–5 drug-related AEs reported were grade 2 insomnia (7/615 [1.1%]) and weight increase (4/615 [0.7%]); there were no grade ≥ 3 drug-related AEs. ^c^The most common grade 2–5 drug-related AE was grade 2 gastroesophageal reflux disease (2/618 [0.3%]); 2 grade 3 drug-related AEs were reported (hypertriglyceridemia (1/618 [0.2%]) and hypercholesterolemia (1/618 [0.2%]). ^d^4 drug-related AEs were reported (flatulence, vertigo, abnormal dreams, and renal impairment). ^e^There were no drug-related serious AEs. ^f^2 non–drug-related fatal serious AEs were reported (gunshot wound [homicide] and unknown cause)

#### Weight

**Fig. 3:**
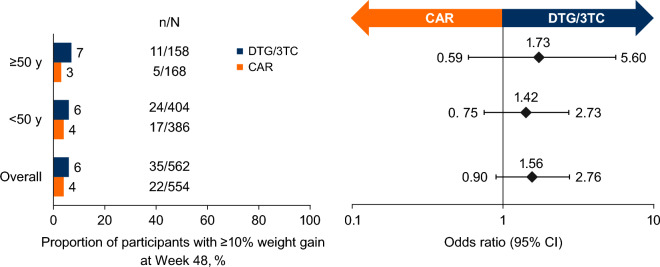
Proportion of participants with ≥ 10% weight gain at Week 48 overall and by age. TANGO and SALSA pooled safety population. *CAR* current antiretroviral regimen, *DTG* dolutegravir, *3TC* lamivudine

Regardless of age category, the DTG/3TC group experienced greater weight gain from baseline at Week 48 than the CAR group, although changes in weight were small. The difference between treatment groups was not significant in participants aged < 50 years (adjusted mean change [SE] for DTG/3TC vs CAR: 1.28 [0.23] vs 1.02 [0.20] kg; treatment difference [95% CI]: 0.27 [− 0.32, 0.86]) but was significant in those aged ≥ 50 years (adjusted mean change [SE] for DTG/3TC vs CAR: 1.28 [0.36] vs 0.03 [0.30] kg; treatment difference [95% CI]: 1.24 [0.33–2.16]). These results were consistent with the overall study results (adjusted mean change [SE]: DTG/3TC, 1.28 [0.19] vs CAR, 0.71 [0.17] kg). Weight gain observed with DTG/3TC was similar between participants aged < 50 vs ≥ 50 years (1.28 kg each), while greater weight increases were observed with CAR in participants aged < 50 vs ≥ 50 years (1.02 vs 0.03 kg, respectively). Among participants aged ≥ 50 years, the difference in weight observed with DTG/3TC vs CAR was significant in female participants (adjusted mean change [SE]: 1.86 [0.61] vs − 0.21 [0.62] kg; treatment difference [95% CI]: 2.08 [0.40–3.75]) but was not significant in male participants (adjusted mean change [SE]: 0.96 [0.45] vs 0.11 [0.35] kg; treatment difference [95% CI]: 0.85 [− 0.26, 1.96]). In the overall pooled population, greater weight gain observed in the DTG/3TC group was mostly driven by outcomes from the SALSA study (adjusted mean change [SE] for DTG/3TC vs CAR: SALSA, 2.01 [0.33] vs 0.64 [0.25] kg; TANGO, 0.82 [0.23] vs 0.77 [0.22] kg; treatment difference [95% CI]: SALSA, 1.37 [0.55–2.19]; TANGO, 0.05 [− 0.57, 0.67]). Within treatment groups, the proportion of participants with ≥ 10% weight gain was low and similar across age groups (DTG/3TC vs CAR: < 50 years, 24/404 [6%] vs 17/386 [4%]; odds ratio [95% CI], 1.42 [0.75–2.73]; ≥ 50 years, 11/158 [7%] vs 5/168 [3%]; odds ratio [95% CI], 1.73 [0.59–5.60]) and consistent with the overall study results (DTG/3TC, 35/562 [6%] vs CAR, 22/554 [4%]; odds ratio [95% CI], 1.56 [0.90–2.76]; Fig. 3).

#### Metabolic parameters

Across both age categories, changes in lipids from baseline to Week 48 were small and generally favored DTG/3TC compared with CAR (Additional file 2). Changes from baseline to Week 48 in plasma/serum renal biomarkers were generally small in both treatment groups within each age group (Additional file 2). A greater decrease from baseline in estimated glomerular filtration rate (eGFR) based on serum cystatin C was observed in participants aged ≥ 50 years in both treatment groups compared with those aged < 50 years (adjusted mean [SE] change from baseline for DTG/3TC vs CAR: < 50 years, 0.812 [0.56] vs 0.003 [0.56] mL/min/1.73 m^2^; ≥ 50 years, − 3.79 [0.93] vs − 4.55 [0.89] mL/min/1.73 m^2^). Regardless of age category, changes in bone biomarkers from baseline to Week 48 were generally small and comparable between treatment groups (Additional file 2).

## Discussion

Overall, pooled findings from the TANGO and SALSA trials demonstrated that DTG/3TC maintained high rates of virologic suppression 1 year after treatment switch with no reported resistance and small and similar changes in CD4 + cell count and CD4 + /CD8 + ratio vs continuing CAR among participants aged ≥ 50 years compared with those aged < 50 years.

Consistent with other studies [14–16], we observed a higher number of concomitant medications used and greater prevalence of comorbidities in participants aged ≥ 50 years. However, this did not impact efficacy and safety outcomes for participants aged ≥ 50 years who switched to DTG/3TC, demonstrating the robust efficacy and safety profile of DTG/3TC regardless of age. In individuals with HIV aged ≥ 50 years, who often have greater concomitant medication use and a higher risk of polypharmacy compared with those aged < 50 years, treatment with a 2-drug vs 3-drug regimen may have the added benefit of limiting the number of medications used, with potentially fewer drug-drug interactions. Few significant drug-drug interaction considerations are noted for DTG/3TC, and it may be an appropriate ART regimen for individuals taking multiple comedications for comorbid conditions as they age [26].

Regardless of age, we observed few AEs leading to withdrawal and SAEs, with comparable incidences between treatment groups. The incidence of SAEs was slightly higher in participants aged ≥ 50 years in both treatment groups, which may be associated with the increased prevalence of age-associated comorbidities observed in this population. As is expected in stable-switch studies such as TANGO and SALSA, drug-related AEs were more frequent in the DTG/3TC group compared with the CAR group across both age groups. Overall AE frequency and intensity were similar across age categories in both treatment groups.

We observed greater weight gain among participants receiving DTG/3TC compared with CAR in both age categories, although the difference was small. In this pooled analysis, this outcome was mostly driven by results from the SALSA study, which may be partly explained by some participants switching from potentially weight-suppressive agents, such as TDF and efavirenz as opposed to TANGO, in which all participants switched from a TAF-based regimen [27–29]. Despite similar absolute weight gain observed with DTG/3TC across age categories, the difference in weight gain between the DTG/3TC vs CAR groups was more pronounced in the ≥ 50 years age group due to lesser weight gain among participants aged ≥ 50 years compared with those aged < 50 years continuing CAR. The difference observed in the CAR group could potentially be due to participants aged ≥ 50 years being more likely to be taking an efavirenz- or TDF-based regimen than those aged < 50 years at study entry as well as having a longer duration of ART use.

Across both age categories, we observed no relevant changes from baseline to Week 48 in renal or bone biomarkers between treatment groups, and directionality of change was similar between age categories. As is expected in a population that is aging [30], decreases in eGFR were slightly greater in the ≥ 50 years age group in both treatment groups. Small changes in lipids generally favored DTG/3TC in both age groups. These results were consistent with 48-week results from the individual TANGO and SALSA studies as well as with a previous study in ART-naive participants [20–22].

There were some limitations to this study, including the relatively small sample of participants aged ≥ 50 and ≥ 65 years. Although the proportion of participants aged ≥ 50 years in TANGO and SALSA (29%) was consistent with the proportion aged ≥ 50 years in the global population of people with HIV (an estimated 24% in 2022) [5], participants aged ≥ 65 years are likely underrepresented (3%). Both studies had a predominantly White and male population, which is not representative of the global population of individuals living and aging with HIV and may limit the generalizability of the results. Furthermore, the heterogeneity of the CAR population in the SALSA study, using a variety of ART regimens, should be considered when interpreting results as switches from different agents may have differential effects.

## Conclusions

Overall, switching to DTG/3TC maintained high rates of virologic suppression and baseline immunologic status among participants aged < 50 and ≥ 50 years, including those aged ≥ 65 years, with no confirmed virologic withdrawals. With a higher prevalence of non-ART medication use and comorbidities associated with age, a well-tolerated 2-drug, single-tablet regimen with robust efficacy such as DTG/3TC may help support the clinical management of individuals with HIV-1 aged ≥ 50 years, particularly those at risk of polypharmacy-driven drug-drug interactions due to evolving health needs and comorbidities.

### Supplementary Information

**Additional file 1.** Adjusted Mean Change From Baseline to Week 48 in CD4 + Cell Count and CD4 + /CD8 + Ratio Overall and by Age: TANGO and SALSA Pooled ITT-E Population. Table showing change from baseline to Week 48 in CD4 + cell count and CD4 + /CD8 + ratio overall and by age

**Additional file 2.** Change from baseline in **A** fasting lipids, **B** plasma/serum renal biomarkers, and **C** bone biomarkers at Week 48 by age: TANGO and SALSA pooled safety population. Figure showing change from baseline to Week 48 in fasting lipids, renal biomarkers, and bone biomarkers by age

## Data Availability

Anonymized individual participant data and study documents can be requested for further research from www.clinicalstudydatarequest.com.

## Abbreviations

AE: Adverse event
ART: Antiretroviral therapy
BMI: Body mass index
CAR: Current antiretroviral regimen
CVW: Confirmed virologic withdrawal
DTG/3TC: Dolutegravir/lamivudine
eGFR: Estimated glomerular filtration rate
FDC: Fixed-dose combination
FTC: Emtricitabine
INSTI: Integrase strand transfer inhibitor
ITT-E: Intention-to-treat exposed
NNRTI: Non-nucleoside reverse transcriptase inhibitor
NRTI: Nucleoside reverse transcriptase inhibitor
PI: Protease inhibitor
SAE: Serious adverse event
TAF: Tenofovir alafenamide
TDF: Tenofovir disoproxil fumarate
