# Supplementary material for: Efficacy and safety of switching to dolutegravir/lamivudine in virologically suppressed people with HIV-1 aged ≥ 50 years: week 48 pooled results from the TANGO and SALSA studies
Source: AIDS Res Ther. 2024 Mar 21;21:17. doi: 10.1186/s12981-024-00604-9 (PMC10958962; doi:10.1186/s12981-024-00604-9)
Supplement: Supplementary file 1 — Additional file 1. Adjusted Mean Change From Baseline to Week 48 in CD4 + Cell Count and CD4 + /CD8 + Ratio Overall and by Age: TANGO and SALSA Pooled ITT-E Population. Table showing change from baseline to Week 48 in CD4 + cell count and CD4 + /CD8 + ratio overall and by age [file 12981_2024_604_MOESM1_ESM.pdf]

Additional file 1. Adjusted Mean Change From Baseline to Week 48 in CD4+ Cell Count and CD4+/CD8+ Ratio Overall and by Age: TANGO and SALSA Pooled ITT-E Population

	**Overall**		**<50 y**		**≥50 y**	
**Parameter**	**DTG/3TC (N=615)**	**CAR (N=619)**	**DTG/3TC (N=438)**	**CAR (N=432)**	**DTG/3TC (N=177)**	**CAR (N=187)**
CD4+ cell count, adjusted mean change (SE), cells/mm^3	22.4 (7.2)	−2.0 (6.9)	29.0 (8.5)	7.6 (8.2)	6.3 (13.6)	−24.7 (12.5)
Adjusted difference (95% CI), cells/mm^3	24.4 (4.9, 43.9)		21.4 (−1.8, 44.6)		30.0 (−5.2, 67.1)	
CD4+/CD8+ ratio, adjusted mean change (SE)	0.037 (0.008)	0.052 (0.009)	0.039 (0.010)	0.048 (0.010)	0.032 (0.016)	0.062 (0.016)
Adjusted difference (95% CI)	−0.015 (−0.039, 0.008)		−0.009 (−0.037, 0.018)		−0.030 (−0.074, 0.013)	

CAR, current antiretroviral regimen; DTG, dolutegravir; ITT-E, intention-to-treat exposed; 3TC, lamivudine.

Adjustment terms included treatment, visit, age, sex, race, baseline value, baseline body mass index, baseline third agent class, treatment-by-visit interaction, baseline value-by-visit interaction, and study, with visit as the repeated factor; subgroup analyses by age were also adjusted for visit-by-age, treatment-by-age, and treatment-by-visit-by-age interactions. For CD4+/CD8+ ratio, baseline CD4+ cell count was an additional adjustment term.
