# Supplementary material for: Efficacy and safety of switching to dolutegravir/lamivudine in virologically suppressed people with HIV-1 aged ≥ 50 years: week 48 pooled results from the TANGO and SALSA studies
Source: AIDS Res Ther. 2024 Mar 21;21:17. doi: 10.1186/s12981-024-00604-9 (PMC10958962; doi:10.1186/s12981-024-00604-9)
Supplement: Supplementary file 2 — Additional file 2. Change from baseline in A fasting lipids, B plasma/serum renal biomarkers, and C bone biomarkers at Week 48 by age: TANGO and SALSA pooled safety population. Figure showing change from baseline to Week 48 in fasting lipids, renal biomarkers, and bone biomarkers by age [file 12981_2024_604_MOESM2_ESM.pdf]

Additional file 2. Change from baseline in **(A)** fasting lipids, **(B)** plasma/serum renal biomarkers, and **(C)** bone biomarkers at Week 48 by age: TANGO and SALSA pooled safety population. Treatment ratio (lipids) or treatment difference (renal and bone biomarkers) (95% CI) shown above or below bars. BMI, body mass index; CAR, current antiretroviral regimen; DTG, dolutegravir; eGFR, estimated glomerular filtration rate; HDL-C, high-density lipoprotein cholesterol; LDL-C, low-density lipoprotein cholesterol; 3TC, lamivudine. Adjustment terms included treatment, visit, age, sex, race, baseline value (log-transformed for lipids), baseline third agent class, baseline CD4+ cell count, treatment-by-visit interaction, baseline value (log-transformed for lipids)-by-visit interaction, and study, with visit as the repeated factor; subgroup analyses by age were also adjusted for visit-by-age, treatment-by-age, and treatment-by-visit-by-age interactions. [a]For renal biomarkers, baseline BMI, diabetes, and hypertension were additional adjustment terms. [b]For bone biomarkers, baseline BMI, smoking history, and vitamin D use were additional adjustment terms.

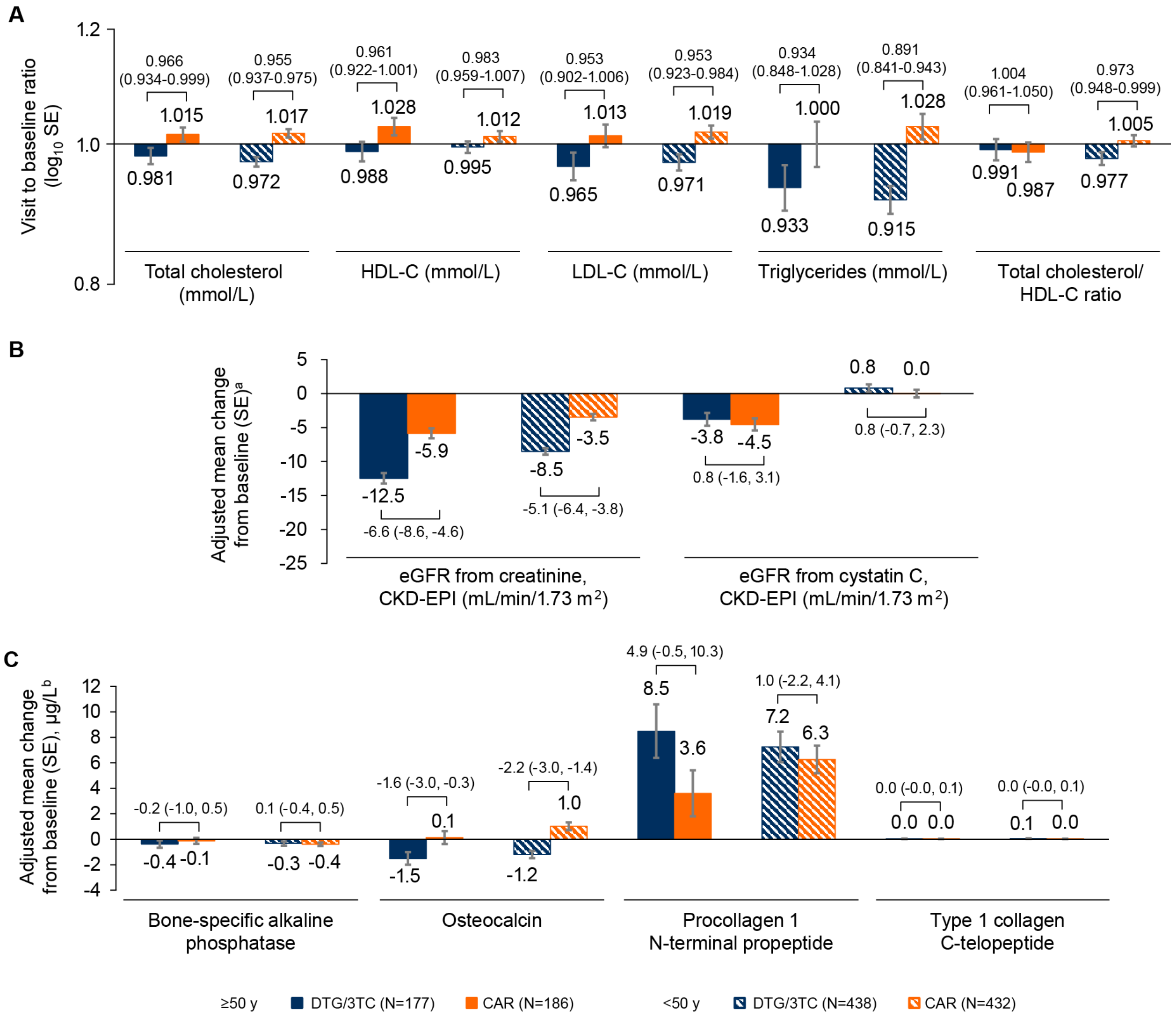

A
Visit to baseline ratio (log10 SE)
1.2
1.0
0.8
0.966 (0.934-0.999)
0.955 (0.937-0.975)
0.961 (0.922-1.001)
0.983 (0.959-1.007)
0.953 (0.902-1.006)
0.953 (0.923-0.984)
0.934 (0.848-1.028)
0.891 (0.841-0.943)
1.004 (0.961-1.050)
0.973 (0.948-0.999)
1.015
1.017
1.028
1.012
1.013
1.019
1.000
1.028
1.005
0.981
0.972
0.988
0.995
0.965
0.971
0.933
0.915
0.991
0.987
0.977
Total cholesterol (mmol/L)
HDL-C (mmol/L)
LDL-C (mmol/L)
Triglycerides (mmol/L)
Total cholesterol/ HDL-C ratio
B
Adjusted mean change from baseline (SE)[a]
5
0
-5
-10
-15
-20
-25
-12.5
-5.9
-8.5
-3.5
-3.8
-4.5
0.8
0.0
-6.6 (-8.6, -4.6)
-5.1 (-6.4, -3.8)
0.8 (-1.6, 3.1)
0.8 (-0.7, 2.3)
eGFR from creatinine, CKD-EPI (mL/min/1.73 m2)
eGFR from cystatin C, CKD-EPI (mL/min/1.73 m2)
C
Adjusted mean change from baseline (SE), µg/L[b]
12
10
8
6
4
2
0
-2
-4
-0.2 (-1.0, 0.5)
0.1 (-0.4, 0.5)
-1.6 (-3.0, -0.3)
-2.2 (-3.0, -1.4)
4.9 (-0.5, 10.3)
1.0 (-2.2, 4.1)
0.0 (-0.0, 0.1)
0.0 (-0.0, 0.1)
-0.4 -0.1
-0.3 -0.4
-1.5
0.1
-1.2
1.0
8.5
3.6
7.2
6.3
0.0 0.0
0.1 0.0
Bone-specific alkaline phosphatase
Osteocalcin
Procollagen 1 N-terminal propeptide
Type 1 collagen C-telopeptide
≥50 y
DTG/3TC (N=177)
CAR (N=186)
<50 y
DTG/3TC (N=438)
CAR (N=432)
